# Attitudinal Change in Elderly Citizens Toward Social Robots: The Role of Personality Traits and Beliefs About Robot Functionality

**DOI:** 10.3389/fpsyg.2015.01701

**Published:** 2015-11-20

**Authors:** Malene F. Damholdt, Marco Nørskov, Ryuji Yamazaki, Raul Hakli, Catharina Vesterager Hansen, Christina Vestergaard, Johanna Seibt

**Affiliations:** ^1^Department of Philosophy and the History of Ideas, School of Culture and Society, Aarhus UniversityAarhus, Denmark; ^2^Unit for Psychooncology and Health Psychology, Department of Oncology, Aarhus University Hospital and Department of Psychology & Behavioural Science, Aarhus UniversityAarhus, Denmark; ^3^Hiroshi Ishiguro Laboratories, Advanced Telecommunications Research Institute InternationalOsaka, Japan

**Keywords:** social robots, attitudes toward social robots, personality, anthropomorphism, human–robot interaction

## Abstract

Attitudes toward robots influence the tendency to accept or reject robotic devices. Thus it is important to investigate whether and how attitudes toward robots can change. In this pilot study we investigate attitudinal changes in elderly citizens toward a tele-operated robot in relation to three parameters: (i) the information provided about robot functionality, (ii) the number of encounters, (iii) personality type. Fourteen elderly residents at a rehabilitation center participated. Pre-encounter attitudes toward robots, anthropomorphic thinking, and personality were assessed. Thereafter the participants interacted with a tele-operated robot (Telenoid) during their lunch (c. 30 min.) for up to 3 days. Half of the participants were informed that the robot was tele-operated (IC) whilst the other half were naïve to its functioning (UC). Post-encounter assessments of attitudes toward robots and anthropomorphic thinking were undertaken to assess change. Attitudes toward robots were assessed with a new generic 35-items questionnaire (attitudes toward social robots scale: ASOR-5), offering a differentiated conceptualization of the conditions for social interaction. There was no significant difference between the IC and UC groups in attitude change toward robots though trends were observed. Personality was correlated with some tendencies for attitude changes; Extraversion correlated with positive attitude changes to *intimate-personal relatedness* with the robot (*r* = 0.619) and to *psychological relatedness* (*r* = 0.581) whilst Neuroticism correlated negatively (*r* = -0.582) with *mental relatedness* with the robot. The results tentatively suggest that neither information about functionality nor direct repeated encounters are pivotal in changing attitudes toward robots in elderly citizens. This may reflect a cognitive congruence bias where the robot is experienced in congruence with initial attitudes, or it may support action-based explanations of cognitive dissonance reductions, given that robots, unlike computers, are not yet perceived as action targets. Specific personality traits may be indicators of attitude change relating to specific domains of social interaction. Implications and future directions are discussed.

## Introduction

Roboticists envisage that by 2020 robotics technology will “influence every aspect of work and home.”^1^ According to official projections, by 2025 the market value of robotics will expand to several trillion US$ per year, mainly due to social robotics, which will be outperforming industrial robotics by a large margin.^2^

Despite these advances the vast majority of residents in the European Community (87% of 26.751 respondents; Public Attitudes Towards Robots, 2012; Special Eurobarometer 382) has of yet no personal experience with robots (e.g., robotic vacuum cleaners or industrial robots) but report positive attitudes toward robot technologies (70%). However, this positive attitude is relative to the specific context in which the robot is applied, as 60% believe robots should be *banned* from being used as caretakers for children, elderly and disabled people, and 69% would feel uncomfortable having their dog being walked by a robot. In line with this only 3% believe robots *should* be used for education or caretaking of children, elderly or disabled people. This illustrates the challenges that may arise when robots are introduced into the social sphere and assigned assistive functions in direct interaction with humans.

Several studies support that a specific negative attitude–where ‘attitudes’ are defined as “the relatively enduring organization of beliefs, feelings, and behavioral tendencies” (Vaughan and Hogg, 2005, p. 150)–pertains to so-called ‘social’ robots, and their applications (Nomura et al., 2006, 2008). Among the numerous factors that may determine or affect these attitudes are gender (Nomura et al., 2006; Schermerhorn et al., 2008; Kuo et al., 2009), cultural background of the participants (Bartneck et al., 2005, 2007; Nomura et al., 2008), age (Bumby and Dautenhahn, 1999; Kuo et al., 2009; Heerink, 2011; Smarr et al., 2012), initial attitude (Stafford et al., 2014), and previous experience with robots (Nomura et al., 2006; Bartneck et al., 2007). Furthermore, attitudes and assumptions about robots may be determined by their design, as for instance zoomorphic robots give rise to the assumption of pet like functionalities (Nomura et al., 2008) whilst more humanlike features give rise to attribution of human-like capabilities (Nomura et al., 2008; Schermerhorn et al., 2008). Likewise, it appears that the more human features the robot possesses, the greater the expectations (Nomura et al., 2008). This may suggest that the expectation of autonomous function is borne out of a more humanoid robot design. Yamaoka et al. (2007) explored what happens if the expectation of autonomy in a humanoid robot is challenged by explicitly informing participants that a robot is tele-operated, when in fact it is autonomous. Regardless of the information given beforehand, 2/3 of participants felt that they were interacting with an autonomous robot (Yamaoka et al., 2007). As pointed out by the authors this could indicate that the participants became so immersed in the communication that they failed to retain the information about the robot. Several studies have explored how presumptions about a robot’s autonomy can be influenced by information about the robot’s functionality; this has mainly been investigated by using the so-called ‘Wizard of OZ paradigm’ in which participants are deceived to believe that a robot is autonomous when in fact it is tele-operated to some degree (for a review see, Riek, 2012). However, so far it has not been explored in which way *attitudes toward robots* change if participants are given truthful information about a robot being tele-operated, or are given no information at all about the degree of autonomy.

The aforementioned investigations may be pivotal to determining the mechanisms for attitude change in this particular area of technology. So far it is not well-understood whether, and to what extent, attitudes toward robots can be influenced. Wu et al. (2014) recently reported that attitudes and acceptance toward assistive robots were unchanged despite several encounters with the robots in healthy elderly and elderly with mild cognitive impairment. The lack of change was attributed to social stigma and uneasiness toward technology (Wu et al., 2014). Conversely Stafford et al. (2010) report more positive attitudes toward a healthcare robot amongst elderly residents at a retirement home after interaction with it (Stafford et al., 2010). Although several studies report positive attitudes toward robots after personal encounters (Mirnig et al., 2012; Yamazaki et al., 2012, 2014) most of these studies do not assess pre-encounter baseline attitudes. Hence, it is difficult to infer whether personal encounters *per se* affect attitudes toward robots or whether, for instance, a selection bias may affect the results, i.e., people with more positive attitudes toward robots at baseline volunteer to partake in the studies. Furthermore, due to the lack of baseline assessments, these studies offer little insight into attitude change.

Determining whether attitude change occurs after encounters with robots and identifying variables that impact such changes are important, especially as more positive attitudes might lead to greater acceptance of robot technology (Ezer et al., 2009). One variable that could potentially influence persistence or change of attitudes is personality. Whilst several studies have explored whether the *robot’s* personality has any effect on the human user’s attitudes toward robots, e.g., by matching between robot-user personalities (Goetz et al., 2003; Lee et al., 2006; Syrdal et al., 2007; Brandon, 2012; Aly and Tapus, 2013; Tay et al., 2014), few have studied the extent to which *user’s* personality affects attitudes toward technology (Cassell and Bickmore, 2003; Luczak et al., 2003). In relation to the latter participants with extravert personality traits appear to have an increased likelihood of responding to technology in a social manner (Luczak et al., 2003) and an increased tendency to ascribe personality to robots with a mechanical or basic appearance, as compared to participants with more introvert personalities (Walters et al., 2007). Conversely, people with high trait Neuroticism and low Extraversion scores preferred the robot to have a more mechanical appearance (Walters et al., 2007). Furthermore, personality may impact proximity behaviors toward robots, since a high score on agreeableness was shown to correlate with a tendency to move closer to robots whilst a high score on neuroticism correlates with a tendency to physically distancing oneself from robots (Takayama and Pantofaru, 2009). This illustrates how personality traits manifest themselves in explicit behaviors toward robots. The aforementioned studies mainly pertain to studies focused on younger participants and though personality is stable in middle and old age (Roberts and DelVecchio, 2000) the effect of personality on change in attitudes toward robots in elderly populations is as of yet unexplored.

Given that elderly citizens are a particular target user group of social robotics, the current state of the art on attitude research in this area thus calls for more detailed investigation. In particular, so far it is unclear whether attitudinal change in elderly people vis-a-vis other kinds of technology, e.g., computers, translates to the very special case of social robots whose design exploits implicit processes of social cognition. Previous studies on age-related differences in attitude change toward computers showed that “although there were no age differences in overall attitudes, there were age effects for the dimensions of comfort, efficacy, dehumanization, and control” (Czaja and Sharit, 1998). While elderly people can change their attitudes toward computers (Jay and Willis, 1992), both of these studies, as well as others (Igbaria, 1993; Mitra et al., 1999), emphasized that these attitudinal changes depend more on the type of information and training interaction with the computer and less on the temporal duration of the experience. Attitudes toward computer technology in elderly can be changed in the course of 3 days (Czaja and Sharit, 1998) and perhaps also in shorter periods, since attitudinal change in general can occur within minutes (Harmon-Jones and Harmon-Jones, 2002; Harmon-Jones et al., 2009). In short, extant research on attitudinal change on computer technology suggests, first, that elderly users of technology present a sufficiently distinct subgroup, as far as base level attitudes are concerned, to warrant separate investigation; second, attitudinal changes can occur also in elderly people during short temporal periods; and third, changes in attitudes toward computer technology were produced by information and practical interaction. These three insights motivated the basic set-up of our pilot study on change of attitudes toward robot technology in elderly people.

As the term is understood in current research, attitudes have three components: cognitive, affective, and behavioral. Following the set up of previous work on change of attitudes toward computer technology we investigated changes in the first two components, cognitive and affective. An attitude thus can change in two ways—if the emotional involvement changes in degree and kind, or if the conceptual content of the attitude changes. Since attitudes toward social robots involve rather subtle and complex cognitive and affective contents (ascriptions of consciousness, self-consciousness, moral agency, moral patiency, etc.) an assessment of changes in attitudes is best undertaken in an interdisciplinary setting involving quantitative and qualitative methods, as well as conceptual analysis. The pilot study reported here addressed this particular challenge of interdisciplinarity in order to explore (i) how elderly citizen’s attitudes toward robots are affected by baseline information about the functionality of robots, (ii) whether they change after repeated direct encounters with a robot and, (iii), finally whether certain personality traits facilitate attitude changes.

## Materials and Methods

### Subjects

Participants were residing at Vikaergård (VG) Rehabilitation Centre in Jutland, Denmark. VG offers temporary accommodation and secondary rehabilitation after hospitalization for citizens after disease or injury. Patients may stay at VG for up to 6 weeks.

Inclusion criteria: the participants who were invited to partake in the pilot study were deemed “poor eaters” by trained rehabilitation staff. This was an effort to ensure a homogeneous population who could potentially benefit clinically from the study design.

Exclusion criteria were as follows: (a) diagnosis or suspicion of dementia as indicated by a Mini Mental Status Examination (MMSE) score of 23 or less (Folstein et al., 1975), (b) diagnosis of neurological or neurodegenerative disease, (c) macular degeneration or severe hearing loss, (d) inability to self-feed (as indicated by diseases of mouth or throat or severe motor impairment).

### Procedure

The pilot study was carried out in accordance with the Declaration of Helsinki and the Regional Committee on Health Research Ethics. Eligible participants were invited to partake in the study by staff at VG who also supplied them with written information about the project. Subjects who agreed to participate and signed written informed consent received a baseline assessment consisting of questionnaires and a structured interview. A trained master-student in psychology undertook the assessments under supervision of a trained psychologist (MFD). In the 3 days following the assessment the participant had lunch (20–40 min) in the company of either a tele-operated robot or a member of staff. Their lunches were video recorded. The participants were randomly assigned to one of three conditions: (a) an informed condition (IC; *n* = 7) where the participants were informed that the social robot would be tele-operated, (b) an uninformed condition (UC; *n* = 7) where the participants were not given any information about the functionality of the robot, (c) a control condition (CT) where the participant had lunch in the company of a member of staff. In all randomization conditions the conversations and conversation topics were non-scripted and mainly focused on the food, weather, health, the stay at VG etc. Hence, the conversation topics did not pertain to attitudes toward robots. The lunch was served in the participants’ private rooms at VG. The control condition was canceled due to unforeseen recruitment problems and the participants excluded (*n* = 3).

Finally, the participants received questionnaires and a structured interview 1 week from the baseline assessment. After the encounter the participants were debriefed on the functionality of the robot. The participants were instructed not to discuss the pilot study with other residents at VG as it could impact the recruitment process and contaminate the data.

### The Robot and the Operators

**FIGURE 1:**
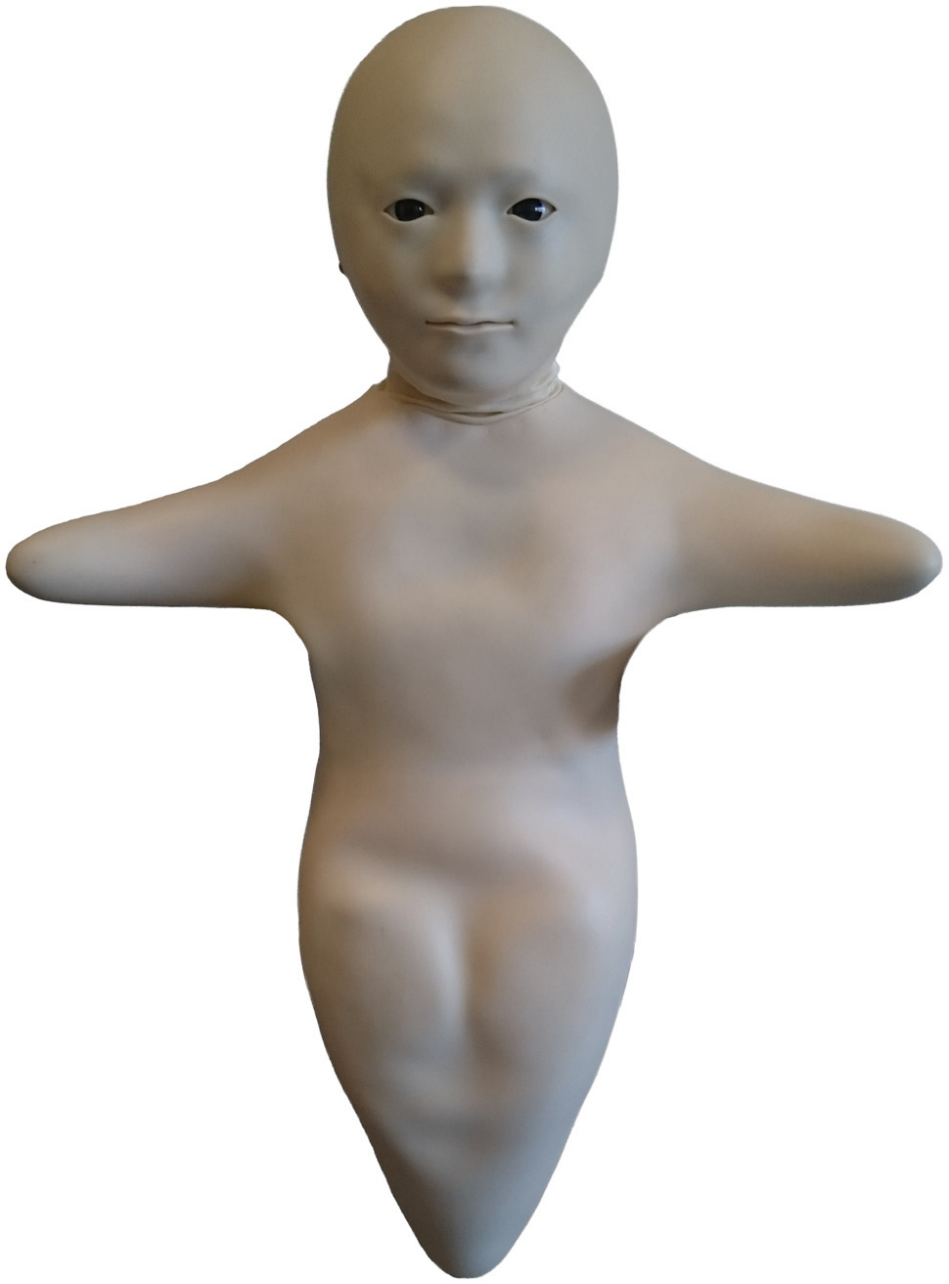
**The Telenoid robot**.

**FIGURE 2:**
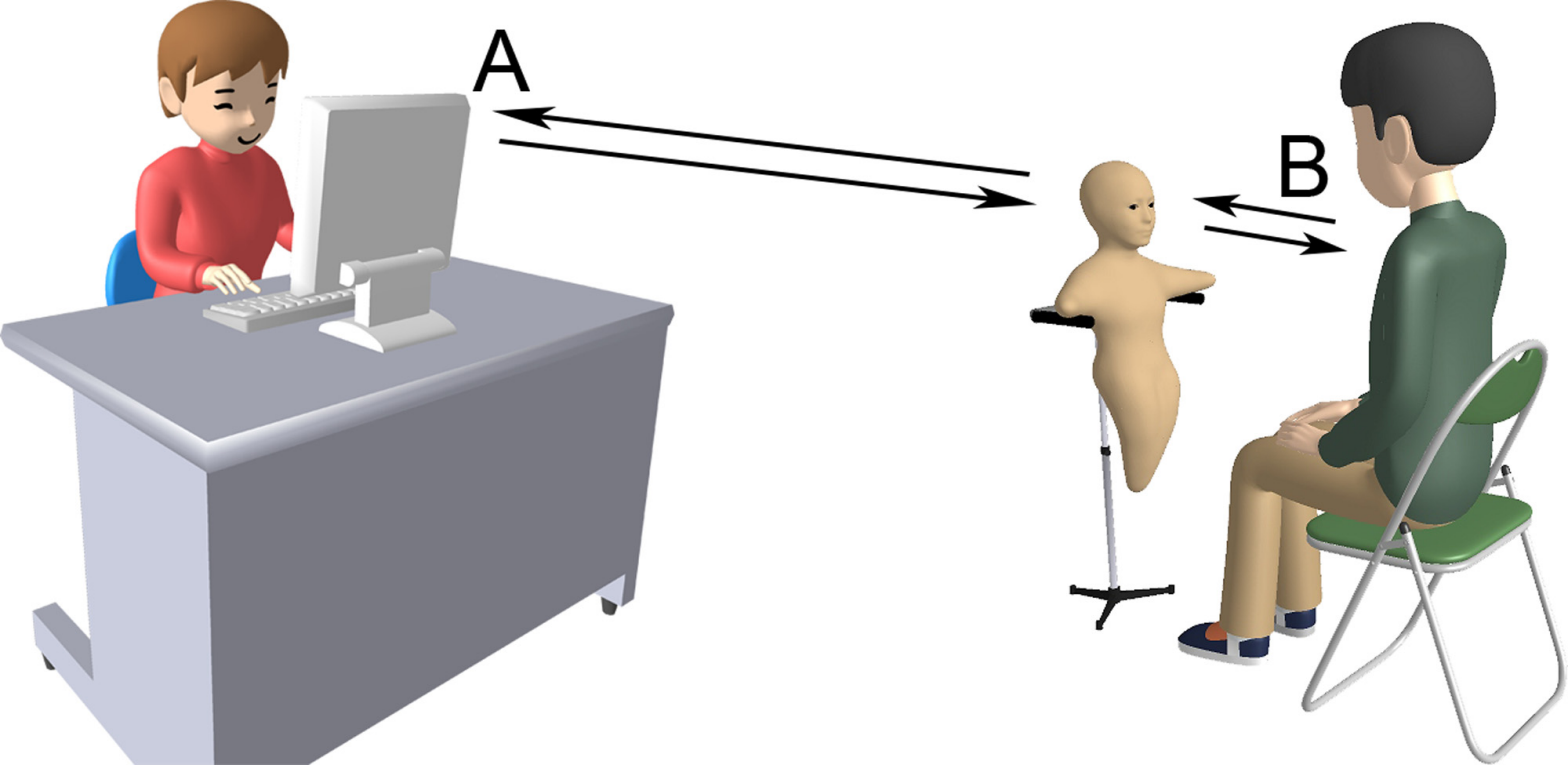
**Simplified visualization of an interaction encounter**.

The Telenoid (see **Figure 1**), a tele-operated android robot developed by Hiroshi Ishiguro from Osaka University and the Advanced Telecommunication Research Institute International, was used. This technology enables two persons, A and B (see **Figure 2**), to communicate with each other using the robot as a communication channel. In contrast to a traditional telephone conversation the interaction facilitated by the Telenoid is asymmetric as the interaction interface is not the same for both parties involved. The operator A controls the robot, which is situated at a different location with the interlocutor B. A’s head movements and voice are simulated by the robot and via a monitor and headset with sensors. A is supplied with a live audio and video feed of the robot’s head and B. The Telenoid’s lip movements follow the speech of A and the robot’s “arms” can be moved in one direction. Furthermore the Telenoid features an idle movement function for the eyes. The basic idea behind this setup is to empower A with a remote embodiment at B’s site via a wireless network connection.

The Telenoid is “designed according to minimum requirements to express humanlike appearance and motion” (Geminoid, n.d.). This neutral design approach is supposed to facilitate B’s free associations with the cues and information provided by A and attempts to avoid any interference imposed by design features such as gender or age.^3^

Three female members of staff, all occupational therapists, were trained in operating the robot. The training contained no specific instruction for conversation content but did contain guidelines of how to reply to questions about robot functionality or personal questions. Overall, the operators were instructed to answer truthfully any questions posed about robot functionality. However, it was not necessary to answer any such questions, as they were not posed. Efforts were made that the participants did not have prior encounters with the operator during their stay at VG, thus would not be able to recognize the operator’s voice in interactions with the robot.

**FIGURE 3:**
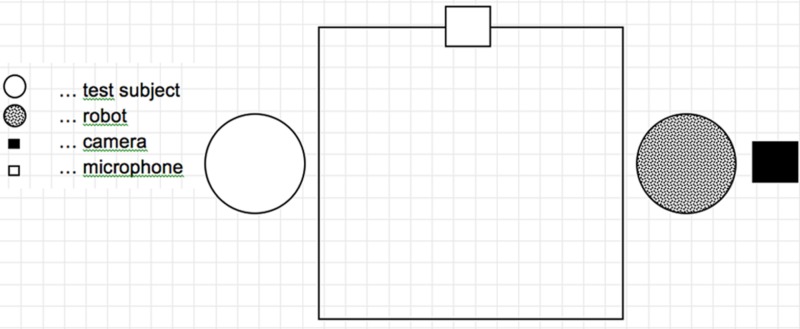
**Schematic layout of the test setting**.

Whilst the participants were getting lunch in the common room the robot, microphone, and camera were set up in the participants’ private room at VG. The camera was mounted on a pole behind the robot overlooking the participant and the lunch table (see **Figure 3**). Thus when the participant returned with their lunch the robot was present on its stand across the table. The robot was controlled from a laptop in an adjacent room with direct video- and audio feed available.

### Measures

#### Demographics and Health

Details on age, marital status, general health, eating habits, depression, and perceived stress were obtained from the participants via questionnaires at baseline. Not all questionnaires are included in the current publication.

#### The NEO-Five Factor Inventory (NEO-FFI)

The NEO-Five Factor Inventory (NEO-FFI; Costa and McCrae, 1992) was used to assess five stable personality dimensions as derived from the five-factor model of personality (NEO-PI-R). The NEO-PI-R is validated cross-culturally (McCrae, 2002) and is available in a validated Danish version. It does not contain items that reflect behavioral, cognitive, or functional well-being of the respondents which would be problematic in the aging study population [for instance the Danish translation of some items in the Tridimensional Personality Questionnaire reads *“I have less energy and I am more tired than most people,” “I believe in luck for the future”* which would not fit the present study given their health status (Cloninger et al., 1991)]. Furthermore, NEO-FFI retains moderate to large correlations with other longer personality questionnaires and has excellent psychometric properties (Larsen, 2007). NEO-FFI was administered at baseline. The questionnaire consists of 60 statements that the respondents rate on a five point Likert scale from “strongly disagree” to “strongly agree.” The items were administered verbally whilst the respondent had the five possible answers available in front of them. The five personality dimensions assessed are: Openness (openness to internal and external stimuli), Conscientiousness (self-discipline and competency), Extraversion (tendency to be sociable and adventurous), Agreeableness (degree of trustfulness, modesty), and Neuroticism (tendency toward experiencing psychological distress or negative affect).

#### The Attitudes toward Social Robots Scale (ASOR-5)

The ASOR-5 questionnaire is a theoretically based, generic scale of attitudes toward social robotics. The questionnaire was developed in an interdisciplinary taskforce consisting of researchers from psychology, anthropology, and philosophy. ASOR-5 consists of the following subscales: (a) Conceptual relatedness (four items), e.g., “*To which degree are you positive about robot technology?*” and “*please describe in three words your impression of the Telenoid from this picture,”* (b) Socio-practical relatedness (eight items), e.g., *“Do you think you would take advice from the Telenoid about which medication you should take?,”* “*Do you think you would be afraid of the Telenoid?*,” (c) Intimate-personal relatedness (five items), e.g., “*Can you imagine having a Telenoid in your own home*?,” “*If you had a Telenoid in your own home would you store it in a broom cupboard?*,” (d) Moral relatedness (five items), e.g., *“Does it matter how people treat robots?,” “Does the Telenoid have a right to electricity?*,” (e) Mental relatedness (five items), e.g., *“Do you think the Telenoid can be happy?,” “Do you think the Telenoid can have hobbies and interests?,”* and (f) Psychological relatedness (six items), e.g., “*I think I would feel sorry for the Telenoid if I saw others be cruel to it,” “I think I would be annoyed if the Telenoid interrupted me in a conversation.”* Besides the conceptual relatedness subscale all other items are rated on a five point Likert scale with higher scores indicating more positive attitudes (scores range from 0 to 140). Negative items were reversed before totaling. Furthermore the questionnaire has optional extra items for follow-up assessments (total of 46 items), which are not included in the current publication. The ASOR-5 is integrated in a large validation study alongside the Godspeed questionnaire (Bartneck et al., 2009), the Negative Attitudes to RobotS questionnaire (Nomura et al., 2005), and the AMPH-10 (see below). For further information please contact the authors.

#### Anthropomorphism Questionnaire (AMPH-10)

A 10-items questionnaire was developed to assess anthropomorphic thinking. Unlike existing questionnaires of anthropomorphism (e.g., The IDAQ; Waytz et al., 2010) the majority of items (six in total) pertain anthropomorphic thinking toward inanimate objects, e.g., “*do you feel grateful toward technology such as a car or computer if you feel it has saved you from a dangerous or difficult situation*” or “*would you ever give a name to an everyday item, such as a Television*”? All items were rated on a four point Likert scale from “very unlikely” to “highly likely” with higher scores indicating more pronounced anthropomorphic thinking (maximum score is 40).

### Statistics

The data was analyzed using IBM SPSS Statistics for Macintosh, Version 21.0. (2012; Armonk, NY, USA: IBM Corp). A change score was calculated defined as the difference in ASOR-5 sub-scores from baseline to follow-up. The informed and uninformed conditions were compared on these continuous variables using independent *t*-tests. Paired sample *t*-tests were used to assess changes in the ASOR-5 sub-scores from baseline to follow-up in the informed (IC) and uninformed (UC) condition. The *t*-test is an acceptable statistical approach, even in very small samples (de Winter, 2013). The possible relationship between personality traits and changes or stability in attitudes toward social robotics was explored by Spearman correlations.

Due to the small sample size and exploratory nature of the pilot study Bonferroni corrections for multiple comparisons were not made. Bonferroni adjustments are normally undertaken by dividing the alpha-level by the number of comparisons made in order to reduce the risk of obtaining false positive results as a consequence of multiple analysis of the same data set (Tabachnick and Fidell, 2001). The necessity of Bonferroni corrections are debated and in the present pilot study we opted for reporting the exact alpha-levels and effect sizes (ESs; Rothman, 1990; Feise, 2002). Samples solely relaying on the alpha-level can be misleading, as smaller samples will possess less statistical power to detect a difference. To inform on the strength of the effect, ESs are reported (Cohen’s *d*) where *d* = 0.2 is considered a small ES, *d* = 0.5 is a medium ES, and *d* = 0.8 or above is deemed a large ES (Cohen, 1988). Due to the modest *n* in the present sample effect-sizes are interpreted conjointly with *p*-values.

A total of 17 elderly participants were enrolled in the study. Three participants were excluded as unforeseen recruitment issues forced us to suspend the control condition.

Repeated *t*-test comparisons showed no significant differences from pre-encounter to post-encounter scores on any of the ASOR-5 domains for the total sample (*n* = 14; see **Table 1**). Hence there was no significant difference in attitude scores on any domains from before they meet the robot till after they had been in company with it during lunch, for up to 3 days. However, a moderate ES (*d* = 0.562) was observed on the *Intimate-personal relatedness* domain, which indicates a non-overlap between the two groups of 33% (Sullivan and Feinn, 2012).

**Table 1:** Repeated two-tailed *t*-test comparisons of the ASOR-5 domains.

	Baseline T1	Post-encounter T2	*t*-test	*p*-value	Cohen’s d
	
	Mean *(SD)*	Mean *(SD)*	*t(13)*	*p-value*	*d*
**ASOR-5 Domains**					
SPR	12.43 (3.13)	11.79 (3.75)	0.529	0.606	0.19
IPR	8.21 (3.02)	9.71 (2.27)	-1.9	0.078	0.56
MOR	7.01 (2.04)	6.86 (2.32)	0.224	0.826	0.07
MER	2.79 (3.56)	4.14 (4.07)	-1.24	0.236	0.35
PSR	14.14 (4.57)	13.79 (4.57)	0.340	0.740	0.08
Total scale	43.46 (9.04)	46.23 (4.55)	0.949	0.361	0.39


*SPR, socio-practical relatedness; IPR, intimate-personal relatedness; MOR, moral relatedness; MER, mental relatedness; PSR, psychological relatedness.*

The participants were assigned to either the IC (*n* = 7) or the UC (*n* = 7) group as they were recruited. The IC and UC groups did not differ significantly in terms of gender distribution (men 71.4% in either group) and there was no significant difference between the IC (*M* = 74.83, *SD* = 12.9) and UC (*M* = 75.29, *SD* = 11.7) groups on age [*t*(13) = 0.06, *p* = 0.948].

Independent two-tailed *t*-tests showed no significant difference between the informed and uninformed condition in attitude change scores on any of the ASOR-5 subscales (see **Table 2**). Hence the change in attitude from pre- to post-encounter did not differ significantly between the two groups who were given different information about robot functionality. However, there was a near significant difference on the socio-practical relatedness subscale where participants who where uninformed about the functionality of the robot, rated it more negatively after meeting it. This is supported by a very large ES (*d* = 1.09), which means that there is a 55% non-overlap between scores in the informed and the uninformed conditions where the latter group was more likely to change their attitude negatively post-encounter.

**Table 2:** Independent *t*-test comparisons of the IC and UC ASOR-5 change scores.

	Informed condition (IC) (*n* = 7)	Uninformed condition (UC) (*n* = 7)	*t*-test	*p*-value	Cohen’s *d*
	
	Mean *(SD)*	Mean *(SD)*	*t(12)*	*p-value*	*d*
**Demographics**					
Age	74.83 (12.9)	75.29 (11.7)	-0.06	0.948	–
**Change scores in ASOR-5 scale^a^**					
SPR	1.57 (4.65)	-2.86 (3.44)	2.03	0.066	1.09
IPR	2.14 (2.04)	0.85 (3.67)	0.81	0.433	0.43
MOR	0.43 (1.72)	-0.71 (2.93)	0.89	0.391	0.48
MER	-0.43 (2.07)	0.29 (0.76)	-1.50	0.160	0.80
PSR	0.14 (2.50)	-0.86 (5.18)	0.46	0.653	0.25
Total scale	3.86 (9.26)	1.50 (12.63)	0.39	0.714	0.21


*^a^Positive scores reflect positive mean changes in subscale measures after meeting the robot. Subscale change scores = subscale score at time 2 – subscale score at time 1. SPR, socio-practical relatedness; IPR, intimate-personal relatedness; MOR, moral relatedness; MER, mental relatedness; PSR, psychological relatedness.*

Spearman correlation analyses were employed to explore possible correlations between attitude change scores on the ASOR-5 questionnaire and personality traits as measured by NEO-FFI. To increase statistical power the IC and UC groups were combined for this analysis. There were significant moderate-high positive correlations between Extraversion (*M* = 30.36, *SD* = 4.05) and the *intimate-personal relatedness* ASOR-5 subscale, and the *psychological relatedness* ASOR-5 subscale (see **Table 3**). There was a significant negative correlation between Neuroticism (*M* = 19.14, *SD* = 6.22) and the ASOR-5 *mental relatedness* subscale. Conscientiousness (*M* = 30.93, *SD* = 4.16), Agreeableness (*M* = 31.21, *SD* = 5.92), and Openness (*M* = 23.5, *SD* = 6.19) did not correlate significantly with any of the ASOR-5 subscales. Furthermore, there was a significant negative correlation between the ASOR-5 *mental relatedness* subscale and anthropomorphic thinking (*M* = 8.5, *SD* = 5.32).

**Table 3:** Spearman correlations between the ASOR-5 subscale change scores and personality traits (NEO-FFI) and anthropomorphic thinking.

Variables	ASOR-5IPR	ASOR-5PSR	ASOR-5SPR	ASOR-5MOR	ASOR-5MER
Openness	0.009	-0.257	0.270	-0.258	0.067
Conscientiousness	0.080	0.229	0.055	-0.136	0.330
Extraversion	**0.619^∗^**	**0.581^∗^**	0.454	0.511	0.085
Agreeableness	0.134	-0.317	-0.060	-0.165	0.099
Neuroticism	0.479	0.224	0.324	0.281	**-0.582^∗^**
Anthropomorphic thinking	-0.061	-0.249	-0.243	-0.292	**-0.662^∗^**


*^∗^*p* < 0.05. SPR, socio-practical relatedness; IPR, intimate-personal relatedness; MOR, moral relatedness; MER, mental relatedness; PSR, psychological relatedness.*

### Qualitative Analysis of Video Data

#### Method and Set-up

The pilot study also included video recordings of the lunch sessions; the camera was mounted in the stand of the Telenoid, showing the participant frontal, from the point of view of the Telenoid. The lunch sessions took place in the participant’s own room, and the Telenoid was seated at the table when the participant was followed into the room by a staff member of the rehabilitation center carrying the food. The video recordings have been analyzed through content analysis, a method used in both quantitative and qualitative studies to analyze written, verbal, or visual communication messages (Cole, 1988; Elo and Kyngäs, 2007). The material is analyzed through a defined framework, so that it should be possible to reach a result as objective as possible, also with different researchers coding and analyzing the material.

The content analysis was framed by two focus points arrived at deductively from the quantitative analysis of the questionnaires. The quantitative analysis showed a lack of change in attitude toward the robot after interaction, which was surprising when seen in relation to studies showing change in attitude after interaction with computers. For the purpose of this paper it was decided to analyze the video data on two specific aspects: attitudes to the Telenoid during the conversation, especially changes in attitudes over the different sessions, and a focus on specific statements about what it was like to talk to the Telenoid. In this way we seek to add a deeper understanding of some of the interesting findings in the pilot study by triangulating qualitative and quantitative data (Karpatschof, 2010).

#### Selected Results of Content Analysis

In all sessions, with both informed and uninformed participants, the participants greeted the Telenoid with hospitable language, answered questions politely, engaged in normal turn-taking. The conversations followed the schema of a normal exchange during lunch as this would be typical at a rehabilitation center, with the general topics being the food being served; whether the participant was able to eat the food; why the participant was at the rehabilitation center; how it was going with the training sessions; the participant’s family situation; the weather. Despite many of the participants volunteering different personal information which the operator could have pursued, the conversations stuck to the frame of a typical conversation between an occupational therapist and a ‘patient.’

Participants in general expressed pleasure and curiosity about engaging in the conversation with the Telenoid, and despite there being some technical problems (e. g. bad sound, uncontrolled head movements) the participants consistently retained the social norms of polite conversation and tried to remain in contact with the Telenoid. If the Telenoid suddenly worked again, the participants immediately continued to answer questions. Most participants finished up the last session by expressing positive statements of having enjoyed themselves and being positively surprised about the experience of being in the company of a robot.

The content analysis also revealed that while there were many positive statements about talking to the Telenoid during the sessions, there was no distinctive change in attitude toward the Telenoid in the course of the successive sessions. However, a change did happen, but it happened within the first few minutes of each session, and could be clearly observed by comparing the beginnings of sessions 1 and 2. When participants first entered their room, they had never seen the Telenoid before; they were asked to sit at the table directly in front of it. All participants required some help in taking their place at the table and bringing the food along, and they would often discuss ‘it’ with the caretaker helping them. Once they were seated the first time they would either greet the Telenoid with some hesitation, or wait until being greeted and then answer. After the first hesitation the conversation would soon follow normal patterns of conversation. The next time the participant came to eat with the Telenoid, there was a significant change in the initial greeting between the participant and the Telenoid. Often the participant would greet the Telenoid already while entering the room, before he/she was in the view of the Telenoid, or they would greet, as if they were greeting someone they knew, as soon as they were sitting at the table, trying to pick up the conversation from yesterday. They showed obvious signs of familiarity and positivity, smiling, waving, looking directly at the Telenoid and seeking eye contact. In the following excerpts from the video recordings it is shown how the initial greetings change between session 1 and 2.

##### Uninformed male participant #45

First session. The participant is driven in to the table in his wheelchair, he is not really looking at the Telenoid.

T: Hello.

P: Hello.

… … (there is a longer pause while the participant is cutting his food.)

T: What are you having for dinner today?

P: I am having filet mignon.

… … (there is a little discussion about the food and the participant starts eating).

T: Can you hear what I am saying?

P: …what, sorry, yes, I can hear you.

…(the participant looks at the Telenoid while answering, but looks away when it is quiet and continues eating. There is a longer pause).

P: But it is a very quiet companion I have.

T: …(laughs a bit)…It is because she wants to give you time to eat your food.

P: Oh, but that doesn’t matter. It is nice and warm, so it won’t hurt if it cools down a bit, while I am being interrupted.

Last session. The participant is placed at the table. As soon as the carer/helper leaves he says:

P: Hi Sussi (a name he has given the Telenoid in an earlier session).

T: Hi Ole.^4^

P: Well, here we are again. I can hear you loud and clear again. It wasn’t so good yesterday. It is much better. Now you have your own pleasant voice back.

T: That is nice to hear.

##### Informed female participant #48a

First session. P: Hello, hello…(the participant is coming into the room, but still not visible).

T: (no answer).

P: What is your name?…What is your name?…What is your name?

T: (no answer).

P: Can’t you say anything? Yum, It is lovely food I am having. … Can I take a picture of you? (gets her phone). Is it allowed to take a picture of you? … I am taking a picture of you. (continues eating).

…(a carer comes in and tells her that there is something wrong with the sound. After a little while the Telenoid makes a sound)…

P: What are you saying? Are you going to say anything now? I have been excited about talking to you, but you are not answering…(continues eating – this happens several times, about 7 min after she has entered the room, the Telenoid is working again).

T: Hello.

P: Hello. Oh so finally you can say something.

T: The sound came on.

P: Yes, what is your name?

T: What do you think is appropriate?

P: Hmm… Robert.

T: Robert? That is fine.

P: Okay, let’s say that then.

T: I just have to start up. And you are already eating?

D: Yes, thank you. It tastes delicious.

Last session. The participant enters the room and initiates the conversation:

P: Hello Robert.

T: Hi.

P: Hi. So, here we are again.

T: Here we are again, yes.

T: Are things going well?

P: It is yes. It is going really well, I think.

##### Uninformed male participant #48b

First session. The Telenoid says hello as the man is being driven into the room. There is no answer. He looks at the Telenoid as he is getting set at the table, but doesn’t say anything. He begins eating his meal and the Telenoid says:

T: Hello Martin.

P: (looks up in surprise and smiles) Hi. It is nice to see you.

T: What is on the menu?

P: Asparagus soup. And it actually tastes very good. I am not sure about the other stuff… Ham, I think. But I can tell you more about it, when I get to that.

T: That sounds good. (Pause, the participant continues eating). How long have you been here at VG?

P: 2.5 weeks, I think, and I have to be here for 1.5 weeks more.

T: And are you happy about being here?

P: Yes I am. It is actually really nice here. They look after you well, and they are giving me a good training.

T: It sounds like the purpose for coming here has been fulfilled.

P: Yes. That is quite right. Actually it is really nice here, and it is also exciting that I got you as a visitor.

… …(P has some problems with hearing)…

P: (leans forward) Sorry, I can’t hear what you are saying, I have some problems with hearing.

Second session. As the participant is coming in and the food is being set out on the table the Telenoid says:

T: Hallo Martin.

P: (in a loud happy voice)…Hallo! It is lovely to see you again.

…(the carer finishes and walks out and says she won’t disturb)

P: (waves dismissively at the carer and looks at the Telenoid with a smile) No, we can easily handle this, right?

T: Let’s hope the food tastes good today.

P: Yes it is ham I think. It looks good.

Last session. Already as we can hear the participant entering the room, we can hear him shout:

P: Hi!

…(The Telenoid doesn’t answer, the participant sits down)…

P: Hi. (pause). You are not saying anything today. Haven’t you been allowed to…

T: (interrupts) Hi!

P: Hi! Oh, it is good to see you again (P is clearly happy and smiling).

T: Yes, same here. Is there still no food for you?

P: No. But hopefully you have had the electricity you need, so that you are not starving.

T: I have had what I need …(a little laugh in the voice).

…as the session is coming to an end, the participant says:

P: I can’t really eat a lot right now.

T: It doesn’t look like very much. Maybe you can eat a few mouthfuls while we talk.

P: Aahh noo…(The participant hesitates a little, but picks up the fork and takes a little).

P: I can’t really eat anymore, but I will try and eat a few mouthfuls when you say so. Oh no, it is not going so well, I am dropping the food. That is not very good.

T: It is ok with me if you drop your food, that doesn’t matter.

P: No, I know that. I am not shy in front of you anymore, because I know you are just sitting here as a robot, who is supposed to help me, and you are doing that really well. It is nice to have you here to talk to.

These illustrations are representative for a pattern we could observe across 12 participants, both informed and uninformed. In sum, the content analysis of the initial greetings between participants and the Telenoid in the video recordings showed that during the very first encounter in the first session participants were somewhat hesitant in starting the interaction but quickly accustomed themselves to the new situation by turning to social norms of conversation and consistently retained this pattern of interaction throughout the remaining sessions.

## Discussion

To our knowledge this is the first study to assess, for a test population of elderly citizens, change in attitudes toward robots in relation to personality traits as well as taking into account pre- and post-encounter assessments. Overall the pilot study indicates that the elderly participants did not display any statistically significant change in attitude toward robots from pre- to post-encounter. However, a moderate ES (*d* = 0.562) was observed on the *Intimate-personal relatedness* domain, which indicates an effect on this domain. Furthermore, there was no significant difference in attitude change between the participants who were informed about the robot being tele-operated and the participants who were uninformed. The results tentatively suggest that beliefs about robot autonomy and functionality do not significantly impact attitude change toward robots in this population of elderly participants. Participants who were uninformed about the robot functionality at baseline did tend to be more reluctant to rate the robot highly on the socio-practical relatedness scale post-encounter; however, this trend did not reach statistical significance (*p* = 0.066) but the finding is supported by a large ES (*d* = 1.06). Personality was correlated with some changes in attitudes toward robots. There was a moderate correlation between the Extraversion and more positive attitude changes to *intimate-personal relatedness* (*r* = 0.619) and to *psychological relatedness* (*r* = 0.581) whilst Neuroticism and also anthropomorphic thinking correlated negatively (*r* = -0.582) with *mental relatedness*.

The analysis tentatively suggests that the level of information given may impact the way elderly relate to the robot on a socio-practical level as indicated by a large ES on the differences in this domain (*d* = 1.06). Hence, the participants who were uninformed about the robot being tele-operated on average had a negative change in the socio-practical relatedness domain. This domain contains items about whether the Telenoid would be trusted to give pertinent, coherent, and relevant information. It appears that the elderly participants who were uninformed about its functionality were more reluctant to trust the validity of the advice from the Telenoid compared to the informed group. The interpretation of this finding has to be done with caution though as it did not reach statistical significance (*p* = 0.77).

Overall, the results of this pilot study indicate that the influence of information about functionality of robots is negligible for promoting attitude change toward robots in elderly participants. Several explanations may be offered for this finding. As pointed out by Yamaoka et al. (2007) the participants may become so immersed in communication with the robot that they simply forget the information given to them beforehand. However, this explanation does not accommodate our finding that there is no significant or limited attitude changes from baseline to post-encounter. Arguably, if the participants become so engrossed in conversation with the robot one should have expected that their attitudes would have changed in either positive or negative direction from baseline. Rather, it seems that baseline attitudes are largely retained regardless of the level of information or number of personal encounters with a robot. This is supported by Wu et al. (2014) who also reported stability of attitudes toward robots amongst healthy elderly despite repeated encounters with a robot (encounters of 30 min a week for 4 weeks). These findings can be interpreted as an expression of cognitive conservatism where initial attitudes are retained and new information or experiences are poorly integrated with the existing cognitive schema (Piaget et al., 1952). This effect may have been inadvertently nurtured by the design of the study as one of the main assumptions about attitudes and attitude change is that attitudes can either be mainly founded on cognitions or on affect and that emotionally arousing experiences are best at changing affect-based attitudes (whilst cognitively based attitudes are changeable by both feelings toward and knowledge about the attitude object; Edwards, 1990; Edwards and Von Hippel, 1995; Fabrigar and Petty, 1999). It seems likely that attitudes toward robots as social agents are more reliant upon affect, and that attitude changes borne out of social interaction with a robot may also be driven by emotional arousal. Hence, the rational answers given by the elderly participants on questionnaires or in interviews may be qualitatively different from observable emotional attitudes and their changes over time as displayed by the participant during social interaction with the robot.

The personality trait Extraversion was positively correlated to an increased likelihood of high scores on intimate-personal relatedness post-encounter. This is in line with the relationship between Extraversion, positive emotionality and a preference toward social interactions reported in existing literature (Costa and McCrae, 1980). The correlation between Extraversion and attitude change in the present study is limited to the two domains and seems to reflect a wish to satisfy communicative needs. Neuroticism and anthropomorphic thinking at baseline were negatively correlated to attitude changes in mental relatedness to the robot.

Neuroticism is associated with negative emotionality and an inflexible mind-set (Costa and McCrae, 1980). Hence, higher scores on neuroticism and anthropomorphic thinking appear to “lock” the participants into a certain way of mentally relating to the robot blocking the likelihood for change. Most likely these results are produced by different underlying ‘mechanisms’ for participants with high scores on anthropomorphic thinking and for participants with high scores on neuroticism; where the former may from the very beginning relate to the robot *as-if* it were a person with inherent mental capacities and not change this view, the latter will probably be reluctant to mentally relate to the robot under any circumstance.

The present pilot study offers an interdisciplinary field-based study with one-on-one interaction between could-be end users and a social robot with a repeated measures design. In summary the quantitative results tentatively suggest that (i) explicit attitudes of elderly citizens toward robots are not significantly affected by baseline information about robot functionality, (ii) explicit attitudes to robots do not significantly change after repeated personal encounters with a robot, (iii) higher scores on the personality trait Extraversion are correlated with higher likelihood for positive change on the subscales *intimate-personal relatedness* and *psychological relatedness* whilst higher scores on Neuroticism were associated with a reduced tendency to change on the *mental relatedness* scale.

Several limitations should be mentioned. Despite the technical advances in robot technology malfunctions still occurred possibly because of wireless interference from various appliances in use at the rehabilitation center. This meant that the session with the robot was sometimes canceled, delayed or that the robot did not operate properly (e.g., displayed tremor-like movements of the head or in one case was suddenly unresponsive). The exact effect of such experiences on attitudes and attitude change was not taken into account in this pilot study. Future studies should consider assessing how participants experience technical malfunctions. Secondly, it is possible that all participants knew that the robot was tele-operated simply due to its speech and mannerism. We did not explicitly assess the participants’ beliefs about the functionality of the robot post-encounter. However, the near-significant change on the socio-practical domain of the ASOR-5 questionnaire for the uninformed condition combined with a large ES indicates that the instructions at randomization worked (since this near-significant difference may reflect differing attitudes based on the information given about the robot). Thirdly, the study design did not allow for use of the full functionality of the robot. In particular the participants did not hug (hugging being a key feature of the robot’s functionality) or even touch the robot, which may have affected their level of emotional investment in the interaction. The decision not to include tactile stimulation, specifically hugging, stemmed from the original design of the study where some participants had to eat in the company of a member of staff as a control condition. It would have been unethical to demand the staff to hug the participants. Fourthly, the moderate *N* limits the generalizability of the results and the statistical power to detect differences. However, this interdisciplinary pilot study uncovered important trends in the complex relationship between age, attitudes, personality and social robots, which can guide future studies in a larger sample where more complex statistical procedures can be applied.

The interplay between the quantitative and the qualitative results of our study suggest several further implications for future research. Since both Wu et al. (2014) and our pilot study find that older people’s attitudes toward robots are largely stable, while studies on the same age group report changes in attitudes toward computers after similar exposure times (Jay and Willis, 1992; Czaja and Sharit, 1998), it is also important to ask whether this difference might have any implications for competing theories of attitudinal change in general. To be sure, if information about functionality and direct interaction changes elderly people’s attitudes toward computers but not, *mutatis mutandis*, their attitudes toward robots, this may be attributed to the type in interaction involved in each case. On the other hand, one might also argue that the observed stability of attitudes toward robots fits well with recent explanations of attitudinal change as “action-based discrepancy reduction” (Harmon-Jones and Harmon-Jones, 2002; Harmon-Jones et al., 2009). According to this account, attitudinal change occurs to reduce cognitive-affective discrepancies so as to facilitate future actions. Since computers are already entrenched in our socio-cultural practices, we perceive them as agentively relevant and thus may react to discrepancies between pre-interaction attitudes and cognitive and affective states during experience by adjusting the former to unblock decision and action pathways. In contrast, robots do not yet have agentive relevance—they are not yet perceived as items that figure in test subject’s action space and relative to which practical decisions need to be taken, thus the reduction of cognitive-affective discrepancies is practically not yet relevant.

However, in light of the selection of results from our qualitative research as reported in Section “Qualitative Analysis of Video Data” above, another possible explanation of the observed stability of attitudes toward robots is possible. According to the “action-based” explanation of attitudinal change, these processes occur in order to reduce a felt discrepancy among cognitions that carry conflicting action tendencies. More precisely, the reduction of discrepancy occurs to eliminate the negative emotion of dissonance (proximal motivation) and to enable efficient action (distal motivation; Harmon-Jones et al., 2009, p. 128). If no discrepancy in action tendencies is experienced, and if accordingly no emotional dissonance is experienced, on the action-based model there is no reason to change one’s attitudes. Based on the qualitative analysis of the video material of our study precisely this appears to be the case. All participants, both informed and uninformed, very quickly (within a few minutes during the first session) settle on the overall interaction pattern of polite social conversation and return to this style of interaction without hesitation, almost eagerly, during subsequent sessions. The fact that several participants choose to give the Telenoid a first name consolidates the interaction frame of social conversation for the duration of their encounter. Most remarkable perhaps, participants stay with the routines of social conversation even when severe technical problems occur (no sound, uncontrolled head movements of the Telenoid). At no time participants displayed any tendencies to break with the action patterns of social conversation with the Telenoid (e.g., by calling for the caretakers during malfunction, or by ending the session prematurely). In short, the pre-encounter attitudes toward the Telenoid did not have to be corrected since the interaction context did not create any conflicting action tendencies and associated negative emotions.

This explanation would imply that future research on attitudinal change toward social robots cannot use the interaction scenarios that social robots are developed for. Social robots are intentionally designed to engage humans in social interaction patterns, exploiting both explicit and implicit (pre-conscious) “mechanism of social cognition” (Frith and Frith, 2008). Thus mere habituation and increased encounter in everyday social contexts are unlikely to change negative human assessments of social robots. Humans are conditioned to uphold the routines of social interactions precisely because these routines serve the evolutionary function of providing agentive guidance in a large variety of situations where agentive insecurity or conflictedness might otherwise occur. On the assumption that the “action-based” explanation of attitudinal change is on the right track, future experiments on attitudinal change toward social robots thus will need to operate with set ups that involve extraordinary interaction context where genuine conflicts of action-tendencies can arise.

## Conflict of Interest Statement

The authors declare that the research was conducted in the absence of any commercial or financial relationships that could be construed as a potential conflict of interest.

## Footnotes

1 Research Agenda 2020 of EuRobotics, a European research conglomerate of 183 robotics firms.

2 McKinsey Global Institute (2013). Disruptive technologies: Advances that will transform life, business, and the global economy.

3 Further information on technical aspects of the Telenoid can be found at http://www.geminoid.jp/projects/kibans/Telenoid-overview.html (accessed 9 June, 2015).

4 Names are changed.
